# Elevated plasma heparin-binding protein is associated with early death after resuscitation from cardiac arrest

**DOI:** 10.1186/s13054-016-1412-4

**Published:** 2016-08-07

**Authors:** Giuseppe Ristagno, Serge Masson, Marjaana Tiainen, Stepani Bendel, Roberto Bernasconi, Tero Varpula, Valentina Milani, Jukka Vaahersalo, Michela Magnoli, Eberhard Spanuth, Simona Barlera, Roberto Latini, Sanna Hoppu, Ville Pettilä, Markus B. Skrifvars, FINNRESUSCI Study Group

**Affiliations:** 1 Department of Cardiovascular Research, IRCCS – Istituto di Ricerche Farmacologiche “Mario Negri”, Milan, Italy; 2 Department of Neurology, Helsinki University Hospital, Helsinki, Finland; 3 Division of Intensive Care Medicine, Kuopio University Hospital, Kuopio, Finland; 4 Division of Intensive Care Medicine, Department of Anaesthesiology, Intensive Care and Pain Medicine, University of Helsinki and Helsinki University Hospital, Topeliuksenkatu 5, PL 266, 00029 HUS, Helsinki, Finland; 5 DIAneering – Diagnostics Engineering & Research, Heidelberg, Germany; 6 Department of Intensive Care, Tampere University Hospital, Tampere, Finland; 7 Intensive Care, Inselspital, Bern University Hospital, Bern, Switzerland; 8 Australian and New Zealand Intensive Care Research Centre, School of Public Health and Preventive Medicine, Monash University, Melbourne Victoria, Australia

**Keywords:** Cardiac arrest, Heparin-binding protein, Post resuscitation, Outcome

## Abstract

**Background:**

An intense systemic inflammatory response is observed following reperfusion after cardiac arrest. Heparin-binding protein (HBP) is a granule protein released by neutrophils that intervenes in endothelial permeability regulation. In the present study, we investigated plasma levels of HBP in a large population of patients resuscitated from out-of-hospital cardiac arrest. We hypothesized that high circulating levels of HBP are associated with severity of post-cardiac arrest syndrome and poor outcome.

**Methods:**

Plasma was obtained from 278 patients enrolled in a prospective multicenter observational study in 21 intensive care units (ICU) in Finland. HBP was assayed at ICU admission and 48 h later. Multiple organ dysfunction syndrome (MODS) was defined as the 24 h Sequential Organ Failure Assessment (SOFA) score ≥ 12. ICU death and 12-month Cerebral Performance Category (CPC) were evaluated. Multiple linear and logistic regression tests and receiver operating characteristic curves with area under the curve (AUC) were performed.

**Results:**

Eighty-two percent of patients (229 of 278) survived to ICU discharge and 48 % (133 of 276) to 1 year with a favorable neurological outcome (CPC 1 or 2). At ICU admission, median plasma levels of HBP were markedly elevated, 15.4 [9.6–31.3] ng/mL, and persisted high 48 h later, 14.8 [9.8–31.1] ng/mL. Admission levels of HBP were higher in patients who had higher 24 h SOFA and cardiovascular SOFA score (*p* < 0.0001) and in those who developed MODS compared to those who did not (29.3 [13.7–60.1] ng/mL vs. 13.6 [9.1–26.2] ng/mL, *p* < 0.0001; AUC = 0.70 ± 0.04, *p* = 0.0001). Admission levels of HBP were also higher in patients who died in ICU (31.0 [17.7–78.2] ng/mL) compared to those who survived (13.5 [9.1–25.5] ng/mL, *p* < 0.0001) and in those with an unfavorable 12-month neurological outcome compared to those with a favorable one (18.9 [11.3–44.3] ng/mL vs. 12.8 [8.6–30.4] ng/mL, *p* < 0.0001). Admission levels of HBP predicted early ICU death with an AUC of 0.74 ± 0.04 (*p* < 0.0001) and were independently associated with ICU death (OR [95 %CI] 1.607 [1.076–2.399], *p* = 0.020), but not with unfavorable 12-month neurological outcome (OR [95 %CI] 1.154 [0.834–1.596], *p* = 0.387).

**Conclusions:**

Elevated plasma levels of HBP at ICU admission were independently associated with early death in ICU.

**Electronic supplementary material:**

The online version of this article (doi:10.1186/s13054-016-1412-4) contains supplementary material, which is available to authorized users.

## Background

Despite initially successful resuscitation, mortality after cardiac arrest remains high, with more than half of patients not surviving to hospital discharge due to “post-cardiac arrest syndrome” [1, 2]. Beside myocardial dysfunction and evolving brain injury, a prominent pathophysiological process characterizing such a syndrome is the systemic inflammation subsequent to whole-body ischemia/reperfusion [2–6]. Accordingly, the systemic inflammatory response observed after cardiopulmonary resuscitation (CPR) provides evident similarities to sepsis and septic shock, with progression toward circulatory failure and multiple organ dysfunction syndrome (MODS) [4, 5]. Indeed, severe organ dysfunction in two or more organ systems is very common in post-cardiac arrest patients and is associated with early mortality [7].

Heparin-binding protein (HBP), also called azurocidin or cationic antimicrobial protein of 37 kDa, is a multifunctional protein contained within the secretory and azurophilic granules of polymorphonuclear leukocytes, and is rapidly released upon adhesion of leukocytes to endothelial cells. Once released, HBP acts as chemoattractant and activator of monocytes and macrophages, and increases vascular permeability with consequent edema and hypoperfusion [8, 9].

The systemic inflammatory response following resuscitation from cardiac arrest includes leukocyte activation, endothelial injury, and vascular response with vascular leakage; thus, elevation in plasma levels of HBP is expected and might represent a potential prognostic marker [2–4]. Indeed, in a small cohort of cardiac arrest patients, early elevation of HBP after resuscitation predicted organ failure and poor long-term neurological outcome [10]. In the present observational study, we examined HBP plasma levels in a large population of patients resuscitated from out-of-hospital cardiac arrest. Since HBP has been shown to predict circulatory failure and increase risk of death in the critically ill patient [9–12], we hypothesized that high circulating levels of HBP in patients resuscitated from cardiac arrest would be associated with the severity of organ dysfunction and early death.

## Methods

### Study design, setting, and selection of participants

The study was an observational cohort study, in which plasma levels of HBP were assessed in adult patients resuscitated from out-of-hospital cardiac arrest. Patients included in the present study were part of the FINNRESUSCI study, a nationwide prospective observational cohort study conducted in 21 hospitals in Finland between March 1, 2010 and February 28, 2011 and aiming to evaluate post-resuscitation care and outcome of out-of-hospital cardiac arrest [13]. The study was approved by the ethics committee of the Helsinki and Uusimaa Hospital district (FINNRESUSCI TUTKIMUS §10, 20.1.201) in addition to local ethics approvals in six of the World Medical Association Declaration of Helsinki. Informed consent from the patient’s next of kin was obtained for data collection and blood sampling. All cardiac arrest patients in whom blood samples were obtained at intensive care unit (ICU) admission were included in the study. Information on the 12-month neurological outcome was available for 276 patients. Thus, these 276 patients were included in the long-term outcome analysis.

### Data collection, processing, and outcomes

The participating hospitals were a part of the Finnish Intensive Care Consortium (FICC) and used the same electronic data management system and data validation software (Web Validator, Tieto, Helsinki, Finland). Data on study patients were prospectively collected using an internet-based case report form. Pre-hospital data were collected by the paramedics in accordance with the Utstein Guidelines and included: whether the arrest was witnessed or not; the administration of bystander-initiated life support; the time from call to the dispatch center and return of spontaneous circulation (ROSC); and the use of adrenaline. In-hospital care data were collected electronically and comprised the use of vasopressors and induced hypothermia, the Sequential Organ Failure Assessment (SOFA) score, the Acute Physiology and Chronic Health Evaluation (APACHE) II score, and ICU mortality. The condition of MODS was defined as a SOFA score ≥ 12 [14, 15]. More specifically, the SOFA score was reported as worst value during the first 24 hours (h) of ICU care. A specialist in neurology blinded to the management in the ICU contacted patients discharged from the hospital by phone 1 year after cardiac arrest and determined neurological outcome according to the Pittsburgh Cerebral Performance Categories (CPC). We defined 12-month good outcome as CPC 1–2, and 12-month poor outcome as CPC 3–5.

### Methods of measurement

Plasma levels of HBP at ICU admission and 48 hours later were measured blinded to case identity and clinical data. Blood samples were collected into ethylenediaminetetraacetic (EDTA) acid tubes and were kept at room temperature for 30–60 min before being centrifuged at 2200 g for 10 min. Plasma samples were then frozen at -20 °C in each participating hospital, prior to being transferred in frozen form to Kuopio University Hospital, where they were stored at -70 °C. Upon analysis, samples were thawed and divided into aliquots. HBP levels were assayed in a single batch using an enzyme immunoassay from Axis-Shield Diagnostics (Dundee, Scotland), according to manufacturer’s recommendations. Limit of detection is reported to be 5.9 ng/mL. Inter-assay coefficients of variation were measured in 11 replicates and were 11 % at 21 ng/mL and 7 % at 81 ng/mL.

### Statistical analysis

Categorical variables are presented as proportions and continuous variables as median with interquartile range (IQR). Baseline characteristics by outcomes occurrence were investigated with the chi-square test for categorical variables; continuous variables were compared by analysis of variance or by the nonparametric Kruskal-Wallis test for continuous non-normally distributed data. Multivariable linear regression was performed on natural logarithm-transformed HBP values to identify the independent factors at resuscitation influencing inflammatory biomarkers levels at ICU admission and 48 h later. Results of linear regression are reported in terms of percent change (exponential of beta coefficient) and *p* values. Multivariable logistic regression was used to identify factors that were predictors of MODS, ICU mortality, and 12-month poor neurological outcome. Age, sex, and all other variables associated with the outcome in the univariate analysis (*p* < 0.05) were included in the multivariable model. Odds ratios (OR) with the corresponding 95 % confidence interval (CI) were calculated and *p* values were considered statistically significant if they were less than 0.05. For each multivariable logistic model, collinearity and calibration were assessed respectively by value of variance inflaction factor (VIF) and Hosmer-Lemeshow test. The discrimination ability of inflammatory biomarkers was evaluated by receiver operating characteristic curve (ROC) analyses. All statistical analyses were performed with SAS software, version 9.2 (SAS Institute, Inc., Cary, NC, USA).

## Results

The FINNIRESUSCI study included 548 patients [13], whose clinical characteristics are reported in Additional file 1. Among these, informed consent for blood sampling was obtained for 245 patients at the time of ICU admission and for an additional 33 patients prior to subsequent sampling 48 h later, giving a total study sample of 278 patients. For these 278 patients, data were available from 245 patients at ICU admission and from 222 patients 48 h later, due to either death or patient transfer. Eighty-two percent of patients (229 of 278) survived to ICU discharge and 48 % (133 of 276) had favorable neurological outcome (CPC 1 or 2) at 12 months (Table 1). Baseline characteristics and factors influencing ICU survival and 12-month outcome are shown in Table 1. Pre-hospital factors univariately associated with ICU survival were a shockable rhythm, no use of adrenaline, a shorter time to ROSC, and the induction of hypothermia, while those associated with long-term survival and good neurological outcome included also a younger age and a witnessed cardiac arrest (Table 1).

**Table 1:** Baseline characteristics and clinical factors at resuscitation in all patients and between ICU survivors and non-survivors and patients with good and poor outcome at 12 months

	Whole population (n = 278)	ICU survival		12-month neurological outcome°	
		Yes (n = 229)	No (n = 49)	Good (n = 133)	Poor (n = 143)
Age, mean (SD)	63 ± 13	63 ± 12	64 ± 14	60 ± 12^§^	65 ± 13
Sex (male), n (%)	229 (82)	189 (83)	40 (82)	109 (82)	119 (83)
Shockable rhythm, n (%)	180 (65)	163 (71)**	17 (35)	108 (81)^§^	71 (50)
Witnessed cardiac arrest, n (%)	254 (91)	212 (93)	42 (86)	128 (96)^§^	124 (87)
Bystander-initiated BLS, n (%)	158 (57)	133 (58)	25 (51)	82 (62)	76 (53)
Adrenaline used, n (%)	186 (67)	141 (62)**	45 (92)	67 (50)^§^	118 (83)
Time to ROSC in min, mean (SD)	21 ± 11	20 ± 11**	25 ± 10	18 ± 10^§^	24 ± 11
Therapeutic hypothermia, n (%)	202 (73)	173 (76)*	29 (59)	104 (78)	97 (68)

Chi-square test for categorical variables; analysis of variance (ANOVA) for continuous variables

*ICU* intensive care unit, *SD* standard deviation, *BLS* basic life support, *ROSC* return of spontaneous circulation

°Data on 12-month survival/outcome were missing for two patients

*p < 0.05 and **p < 0.01 vs. ICU death; ^§^ *p* < 0.01 vs. poor outcome at 12 months

Normal values for plasma HBP in the general population are approximately 6 ng/mL [16, 17]. In our patients, median plasma levels of HBP were more than twofold, 15.4 [9.6–31.3] ng/mL, at ICU admission and persisted high 48 h later 14.8 [9.8–31.1] ng/mL. Levels of HBP were significantly higher in patients with an initial non-shockable rhythm compared to those with a shockable rhythm, and in the instance of longer time to return of spontaneous circulation (ROSC) (Table 2). By linear regression models, the common independent determinants of levels of HBP were: the presence of a non-shockable rhythm (64 % increase from shockable to non-shockable, *p* < 0.0001 at ICU admission, and 54 % increase at 48 h, *p* = 0.013) and a longer time to ROSC (1.6 % increase for each minute, *p* = 0.009 at ICU admission).

**Table 2:** Heparin-binding protein (HBP) levels by age, cardiac arrest (CA) presenting rhythm, time to ROSC, and induced hypothermia

Variable	n	HBP ng/mL
*Admission levels*		
Age, year		
<59	79	15.5 (9.1–33.7)
59–68	86	15.7 (9.9–30.2)
>68	80	14.5 (9.9–31.5)
*p value*		*0.967*
CA presenting rhythm		
Shockable	154	13.1 (8.9–21.5)
Non-shockable	90	21.5 (11.4–46.1)
*p value*		*<0.0001*
Time to ROSC, min		
1–15	83	11.9 (7.9–21.2)
16–24	83	17.4 (9.9–33.2)
25–57	79	18.4 (10.8–41.7)
*p value*		*0.004*
*48 hour levels*		
Age, year		
<59	75	14.5 (9.7–28.3)
59–68	81	13.5 (9.6–31.1)
>68	66	15.7 (11.7–31.7)
*p value*		*0.619*
CA presenting rhythm		
Shockable	159	14.1 (9.7–26.1)
Non-shockable	63	19.6 (10.0–59.0)
*p value*		*0.023*
Time to ROSC, min		
1–15	77	13.2 (8.2–22.6)
16–24	72	15.0 (11.9–28.5)
25–57	73	17.7 (11.5–34.3)
*p value*		*0.038*
Induced hypothermia		
Yes	175	15.2 (10.5–31.7)
No	47	13.4 (7.7–29.2)
*p value*		*0.086*

Data are reported as median and (interquartile range). Age and time to ROSC are reported as categorical variables divided in their tertiles. *p* value from Kruskal-Wallis test

*ROSC* return of spontaneous circulation

**Fig. 1:**
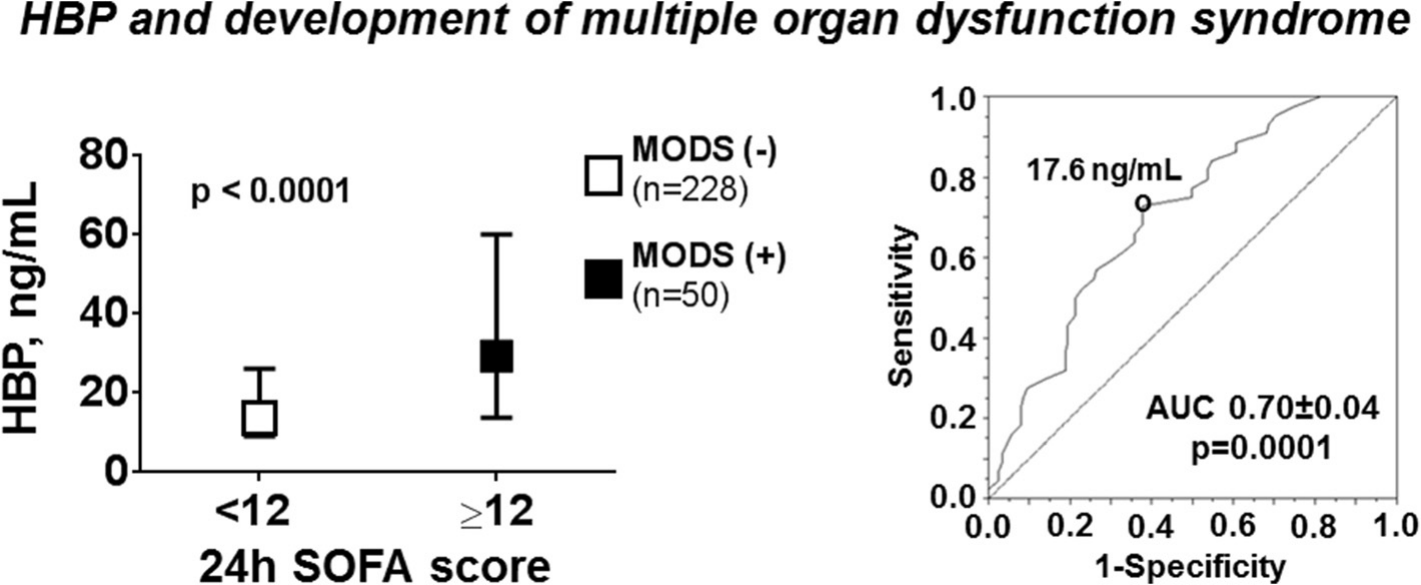
Plasma levels of HBP at ICU admission in patients with or without multiple organ dysfunction syndrome (MODS), and corresponding receiver operating curve (ROC) and area under the curve (AUC). Data are reported as median and interquartile range; *p* value from the Kruskal-Wallis test. *HBP* heparin-binding protein, *SOFA* Sequential Organ Failure Assessment

At ICU admission, plasma levels of HBP were significantly higher in patients with higher 24 h SOFA scores (Additional file 2). Moreover, HBP levels were significantly higher in patients who developed cardiovascular failure (Additional file 2) and in those who developed MODS compared to those who did not (Fig. 1). The area under the curve (AUC) of the ROC curve for discrimination of MODS was 0.70 ± 0.04 (*p* = 0.0001). A plasma level of 17.6 ng/mL of HBP had a balanced sensitivity (0.73) and specificity (0.62) to predict development of MODS (Fig. 1). The odds ratios for prediction of MODS are reported in Table 3.

**Table 3:** Univariate and multivariate logistic models for the prediction of multiple organ dysfunction syndrome (MODS), ICU death, and 12-month poor outcome

	Univariate			Multivariate		
	OR	95 %CI	*p* value	OR	95 %CI	*p* value
MODS						
HBP, 0 hr	1.339	1.023–1.753	0.034	1.193	0.889–1.601	0.240^1^
ICU death						
HBP, 0 hr	1.843	1.280–2.654	0.001	1.607	1.076–2.399	0.020^2^
HBP, 48 hr	1.066	0.726–1.565	0.745	0.939	0.595–1.481	0.786^3^
12-month poor outcome						
HBP, 0 hr	1.980	1.170–3.351	0.010	1.454	0.890–2.375	0.135^3^
HBP, 48 hr	1.337	0.965–1.851	0.081	1.154	0.834–1.596	0.387^3^

OR, odds ratio per 1 SD increase (SD of HBP is 62.67 at 0 hr and 76.14 at 48 hr)

*ICU* intensive care unit, *CI* confidence interval, *HBP* heparin-binding protein, *CA* cardiac arrest, *ROSC* return of spontaneous circulation

Covariates included in the multivariate models are;

^1^CA presenting rhythm (shockable vs. not shockable), use of adrenaline, age, and sex

^2^CA presenting rhythm (shockable vs. not shockable), use of adrenaline, time to ROSC (min), induced hypothermia, age, and sex

^3^CA presenting rhythm (shockable vs. not shockable), use of adrenaline, time to ROSC (min), induced hypothermia, age, and sex

**Fig. 2:**
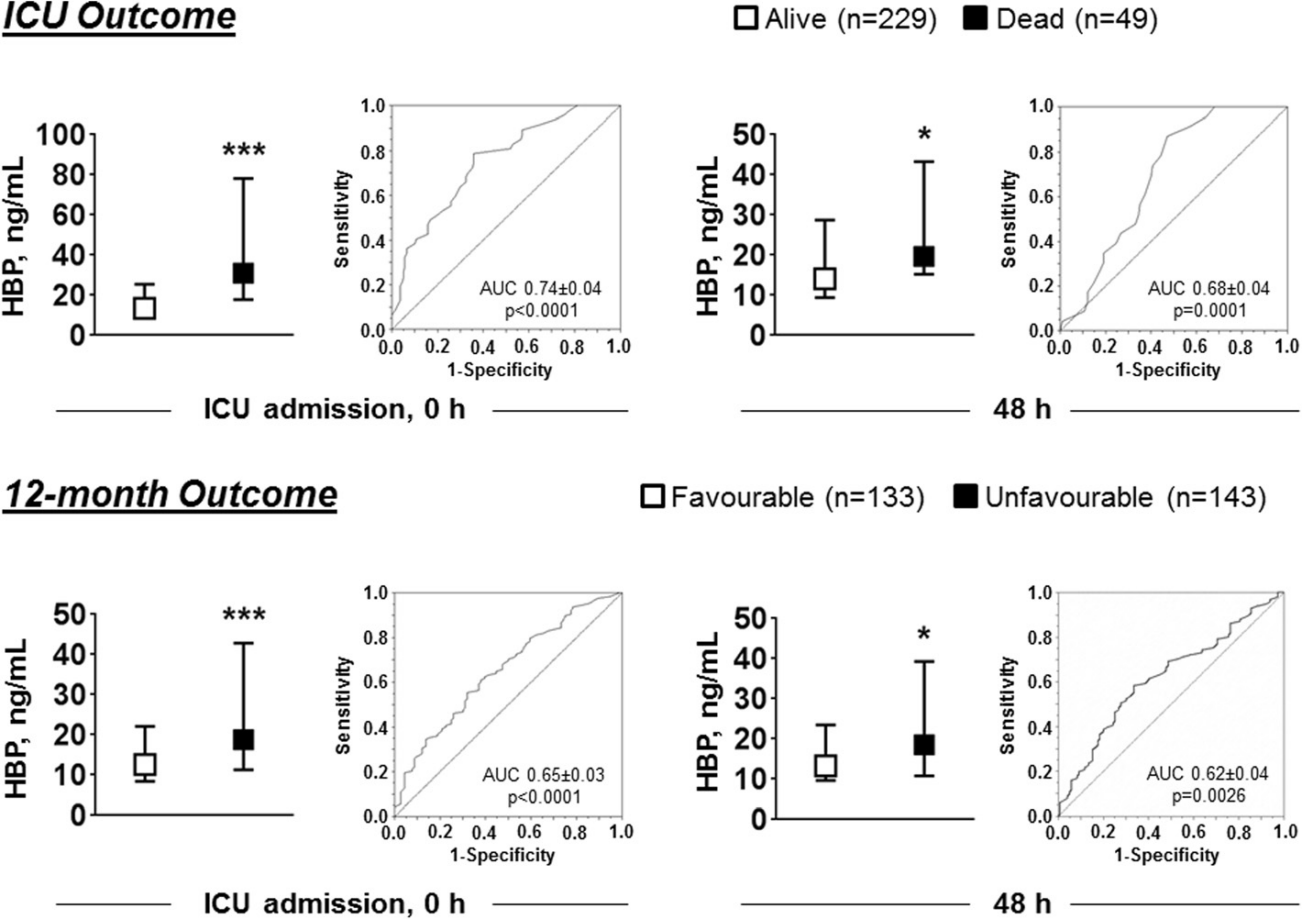
Plasma levels of HBP at ICU admission (0 h) and 48 hour later in ICU survivors and non-survivors (*on the top*) and in patients with favorable and non-favorable 12-month outcome (*on the bottom*), with corresponding receiver operating curves (ROC) curve and area under the curves (AUC). Data are reported as median and interquartile range; *p* value from Kruskal-Wallis test: *p < 0.05; ***p < 0.0001. *HBP* heparin-binding protein, *ICU* intensive care unit

Plasma levels of HBP at ICU admission and 48 h later were significantly higher in patients who died compared to those who survived to ICU discharge (Fig. 2). The odds ratios for prediction of ICU mortality of HBP are detailed in Table 3. In a multivariable model, including age, sex, the initial cardiac arrest rhythm (shockable or non-shockable), time to ROSC, use of adrenaline, and whether induced hypothermia was applied or not (Table 1), plasma levels of HBP at ICU admission were independently associated with ICU death, OR [95 %CI] 1.607 [1.076–2.399] (Table 3). The study had a power of 80 % (at α = 0.05) to detect an effect size of 1.55 (expressed as OR), for 1 SD increase in HBP levels, on ICU death. The AUC of the ROC curve for discrimination of ICU death was 0.74 ± 0.04 (*p* < 0.0001, Fig. 2) and a value of 17.6 ng/mL predicted ICU death with a sensitivity of 0.79 and a specificity of 0.64.

Plasma levels of HBP at ICU admission and at 48 h later were also significantly higher in patients who had a poor 12-month neurological outcome compared to those who had a good recovery (Fig. 2). However, in the multivariable model, HBP was not independently associated with 12-month poor outcome (Table 3). The AUC of the ROC curve for discrimination of 12-month poor outcome was 0.65 ± 0.04 (*p* < 0.0001, Fig. 2). The odds ratios for prediction of 12-month poor outcome at ICU admission and 48 h later are detailed in Table 3.

At ICU admission, serum lactate levels were also recorded in 72 % of patients. When compared, no difference between the AUCs of HBP and those of lactate for discrimination of MODS, ICU death, and poor 12-month outcome (*p* = 0.36, *p* = 0.99, and *p* = 0.97, respectively) were observed, as described in Additional file 3.

## Discussion

The present study suggests that plasma levels of HBP rise early in patients resuscitated from cardiac arrest. Indeed, higher plasma levels of HBP at ICU admission were associated with higher 24 h SOFA score and development of MODS and were independently associated with death in ICU. Plasma levels of HBP were also significantly higher in patients with an unfavorable 12-month neurological outcome compared to those with a favorable outcome.

HBP is a granule protein mainly derived from neutrophils and is released from both secretory vesicles and azurophilic granules [8, 18]. Secretory vesicles release HBP rapidly upon cross-linking of β2 integrins on the surface of neutrophils, while azurophilic granules release HBP more slowly. Whole-body ischemia during cardiac arrest leads to endothelial activation and systemic inflammation, and neutrophils are a determinant in the early step of the inflammatory process [19]. As early as 3 h after resuscitation, blood concentrations of soluble intercellular adhesion molecule-1, soluble vascular cell adhesion molecule-1, and P- and E-selectins increase, suggesting leukocyte and endothelial activation, which may account for a rapid HBP release [4, 20, 21]. In our study, the median HBP levels at ICU admission were already twofold compared to normal values. A potential role of HBP in the pathogenesis of post-resuscitation syndrome cannot be excluded since HBP is known to induce cytoskeletal rearrangement of endothelial cells with subsequent breakdown of cell barriers and increases of the macromolecular efflux [8, 18]. Thus, HBP plays a central role in endothelial permeability regulation, increasing vascular permeability and leading to edema, hypoperfusion, tissue hypoxia, and organ dysfunction [8, 22].

Our study confirms earlier results obtained from 84 patients resuscitated from cardiac arrest, in whom an early elevation of HBP, i.e., at 6 h and 12 h after resuscitation, predicted organ failure and poor 6-month neurological outcome [10]. In that study, HBP levels at 6 h yielded an AUC value of 0.68 for discrimination of long-term poor neurological outcome, which is close to the moderate AUC of 0.65 observed in our cohort. However, in contrast to early results, at the multivariate analysis, high HBP levels were not independently associated with 12-month poor outcome in our population. Our study was performed on a population more than threefold greater than Dankiewicz’s study [10] and thereby the results are likely to be more robust. In addition, in our population, HBP performance in predicting MODS, ICU death, and 12-month outcome was similar to that of serum lactate (Additional file 3), which is one of the initial laboratory findings widely used to predict mortality in patients with different critical illnesses, including cardiac arrest [23, 24]. Thus, HBP admission level is associated with outcome, but it fails to emerge as a new prognostic tool superior to other routinely used biomarkers, i.e., lactate.

HBP levels have been reported to be significantly higher in ICU patients with severe sepsis or septic shock compared to patients with a non-septic illness. More specifically, a plasma HBP concentration of ≥ 15 ng/mL was associated with development of severe sepsis and circulatory failure and increased risk of death [11, 12]. In our study, HBP levels were already > 15 ng/mL at ICU admission, with values above 30 ng/mL in patients who subsequently died in the ICU. Indeed, a level of HBP > 17.6 ng/mL at ICU admission was associated with higher risk of developing MODS, and with a 50 % higher risk of dying in ICU, compared to patients with lower values.

Earlier studies have also reported that elevated concentrations of other inflammatory mediators, i.e., C-reactive protein or procalcitonin, also correlate with patients’ clinical states and predicted poor outcome after cardiac arrest [14, 25]. Circulating concentrations of those biomarkers, however, rise slowly, achieving values predictive of outcome only after 12 to 24 hours following resuscitation. In our study, patients exhibiting high HBP already at ICU admission subsequently developed a more severe shock and were more likely to die in ICU. Our results resemble those of earlier reports in septic patients, indicating HBP as the best early predictor of vascular failure and risk of developing circulatory failure and organ dysfunction, when compared to other laboratory parameters, including procalcitonin, interleukin-6 (IL-6), and C-reactive protein [12, 26]. HBP has been now confirmed as an early biomarker that may have a clinical role in the prediction of imminent death after cardiac arrest, similarly to other early inflammatory biomarkers previously assessed by us in the same population, i.e., PTX3 and sST2 [15], but with limited prediction of long-term outcome, in contrast to others, i.e., IL-6 or kynurenine metabolites [6, 27].

Plasma levels of the inflammatory marker HBP were higher in patients with non-shockable rhythm and with longer time to ROSC. As previously suggested, because non-shockable rhythms are usually associated with other pathological conditions, the high levels of inflammatory markers may reflect the presence of a general inflammatory status that preceded and/or led to cardiac arrest [15, 28]. A longer duration of cardiac arrest, including both no-flow and low-flow times, is known to be associated with a greater severity of post-cardiac arrest syndrome, and plausibly accounted for the higher levels of HBP [3, 15, 27, 29].

We acknowledge several limitations in the interpretation of our findings. First, this was a biomarker substudy of the FINNRESUSCI study. Overall, the FINNRESUSCI population was characterized by a high rate of shockable cardiac arrests and a favorable outcome [13]. In addition, some difference between the patients included in this study and those not included were present (Additional file 1). Indeed, patients with blood samples were somewhat less severely ill than those without blood samples, as represented by the significantly greater percentage of shockable rhythms and survival to ICU and hospital discharge and to 1 year with a favorable neurological outcome. Nevertheless, early HBP elevation was consistently observed after resuscitation in patients who presented poor early outcome. In addition, all patients with a blood sample were included in the study and patients had been treated in 21 different ICUs, including academic and non-academic hospitals covering the majority of a single country and thus suggesting generalizability of the results. Furthermore, in the biomarker analyses, data collection, outcome evaluation and statistics were performed independently. Second, we assayed HBP only at two time points after resuscitation. However, previous studies both in sepsis and cardiac arrest have proven the early prognostic role of this protein and more specifically within 6–12 h after hospital admission, with subsequent decreases [10, 26].

## Conclusions

The present study demonstrated in a large population of out-of-hospital cardiac arrests, that elevated plasma levels of HBP at ICU admission were associated with high 24 h SOFA score and were independently associated with early death in ICU.

## Abbreviations

AUC, area under the curve; CI, confidence interval; CPC, Cerebral Performance Category; CPR, cardiopulmonary resuscitation; h, hour; HBP, heparin-binding protein; ICU, intensive care unit; IL-6, interleukin-6; IQR, interquartile range; MODS, multiple organ dysfunction syndrome; OR, odds ratio; ROC, receiver operating curve; ROSC, return of spontaneous circulation; SOFA, Sequential Organ Failure Assessment

## Additional files

Additional file 1: Baseline characteristics and clinical factors in all FINNRESUSCI patients and in patients with blood samples. Description of data: a table reporting the main clinical characteristics of the patients included in the overall FINNRESUSCI trial and the patients included in the present biomarker substudy. (DOCX 14 kb) Additional file 2: Plasma levels of heparin-binding protein (HBP) at ICU admission in relationship to the 24-hour SOFA score and 24-hour cardiovascular SOFA score. Description of data: at ICU admission, plasma levels of HBP were significantly higher in patients with higher 24 h SOFA scores. Moreover, HBP levels were significantly higher in patients who developed cardiovascular failure. (DOCX 19 kb) Additional file 3: Receiver operating characteristic curves of the discrimination value of plasma HBP at intensive care unit (ICU) admission and lactate and multiple organ dysfunction syndrome (MODS), ICU death, and 12-month poor outcome. Description of data: there was no difference in the areas under the curve (AUCs) of HBP and lactate for the discrimination of MODS, ICU mortality or 12-month poor outcome. (DOCX 68 kb)
