# Supplementary material for: Elevated plasma heparin-binding protein is associated with early death after resuscitation from cardiac arrest
Source: Crit Care. 2016 Aug 7;20:251. doi: 10.1186/s13054-016-1412-4 (PMC4976065; doi:10.1186/s13054-016-1412-4)
Supplement: Additional file 1: — Baseline characteristics and clinical factors in all FINNRESUSCI patients and in patients with blood samples. Description of data: a table reporting the main clinical characteristics of the patients included in the overall FINNRESUSCI trial and the patients included in the present biomarker substudy. (DOCX 14 kb) [file 13054_2016_1412_MOESM1_ESM.docx]

**Additional file 1**

**Table.** Baseline characteristics, clinical factors, and outcomes in all FINNRESUSCI patients and in patients with blood samples.

|  | **Whole FINNRESUSCI**  **population**  (n=548) | **Population with blood samples**  (n=278) |
| --- | --- | --- |
| **Age**, mean (IQR) | 63 (54-72) | 63 (56-72) |
| **Sex** (male), n (%) | 380 (75) | 229 (82)** |
| **Shockable rhythm**, n (%) | 281 (51) | 180 (65)** |
| **Witnessed cardiac arrest**, n (%) | 448 (89) | 254 (91) |
| **Bystander initiated BLS**, n (%) | 273 (54) | 158 (57) |
| **Time to ROSC** **in** **min,** mean (SD) | 20 (15-28) | 20 (13-28) |
| **Induced hypothermia**, n (%) | 311 (62) | 202 (73)** |
| **ICU survival,** n (%) | 429 (78) | 229 (82)* |
| **Hospital survival,** n (%) | 298 (54) | 168 (60)** |
| **12-month survival,** n (%) | 235 (43) | 143 (51)** |
| **Good 12-month outcome,** n (%) | 213 (39) | 133 (48)** |

IQR, interquartile range; BLS, basic life support.

* p < 0.05 and ** p < 0.01 vs. whole FINNRESUSCI population
