# Supplementary material for: Elevated plasma heparin-binding protein is associated with early death after resuscitation from cardiac arrest
Source: Crit Care. 2016 Aug 7;20:251. doi: 10.1186/s13054-016-1412-4 (PMC4976065; doi:10.1186/s13054-016-1412-4)

**Additional file 2**

**Plasma levels of heparin binding protein (HBP) at ICU admission in relationship to the 24-hour SOFA score and 24-hour cardiovascular SOFA score.**

Data are reported as median and interquartile range;

p-value is derived from the Kruskal-Wallis test.


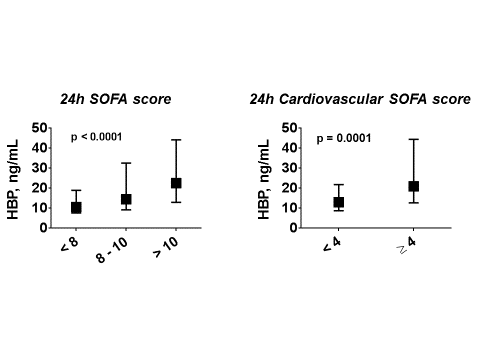

Supplement: Additional file 2: — Plasma levels of heparin-binding protein (HBP) at ICU admission in relationship to the 24-hour SOFA score and 24-hour cardiovascular SOFA score. Description of data: at ICU admission, plasma levels of HBP were significantly higher in patients with higher 24 h SOFA scores. Moreover, HBP levels were significantly higher in patients who developed cardiovascular failure. (DOCX 19 kb) [file 13054_2016_1412_MOESM2_ESM.docx]
