# Supplementary material for: Elevated plasma heparin-binding protein is associated with early death after resuscitation from cardiac arrest
Source: Crit Care. 2016 Aug 7;20:251. doi: 10.1186/s13054-016-1412-4 (PMC4976065; doi:10.1186/s13054-016-1412-4)

**Additional file 3**

**Receiver operating characteristic curves of plasma HBP or serum lactate at intensive care unit (ICU) admission and multiple organ dysfunction syndrome (MODS), ICU death, and 12-month poor outcome.** There was no difference in the areas under the curve (AUCs) of HBP and lactate for the discrimination of MODS, ICU mortality or 12-month poor outcome.


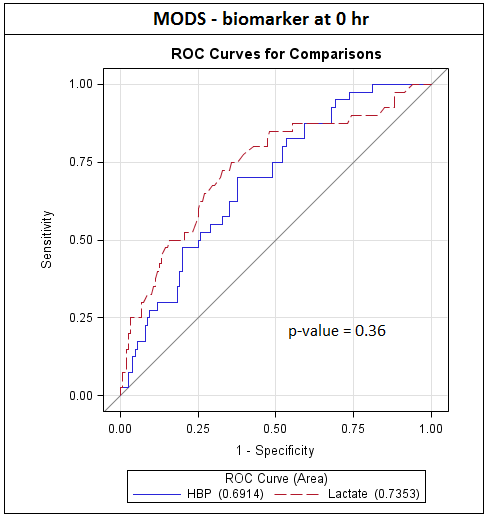


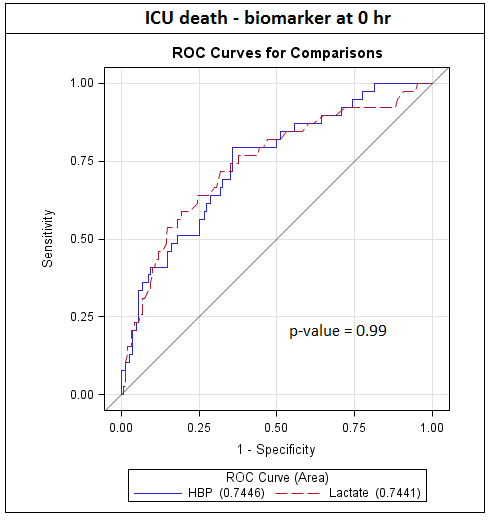


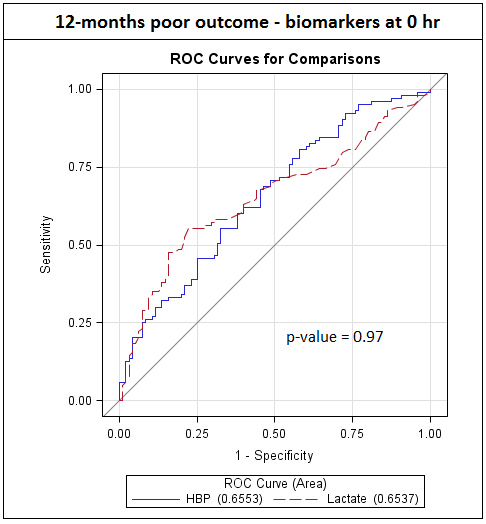

Supplement: Additional file 3: — Receiver operating characteristic curves of the discrimination value of plasma HBP at intensive care unit (ICU) admission and lactate and multiple organ dysfunction syndrome (MODS), ICU death, and 12-month poor outcome. Description of data: there was no difference in the areas under the curve (AUCs) of HBP and lactate for the discrimination of MODS, ICU mortality or 12-month poor outcome. (DOCX 68 kb) [file 13054_2016_1412_MOESM3_ESM.docx]
